# Chlorpyrifos residue level and ADHD among children aged 1–6 years in rural China: A cross-sectional study

**DOI:** 10.3389/fped.2022.952559

**Published:** 2022-10-14

**Authors:** Wenjuan Zhou, Yuanying Deng, Chen Zhang, Hongmei Dai, Lan Guan, Xiangwen Luo, Wei He, Jing Tian, Lingling Zhao

**Affiliations:** ^1^ Department of Pediatrics, Third Xiangya Hospital, Central South University, Changsha, China; ^2^ School of Public Health, Central South University, Changsha, China; ^3^ Hunan Academy of Agricultural Sciences, Changsha, China

**Keywords:** chlorpyrifos, ADHD, micronutrients, vitamin D, cross-sectional study, children

## Abstract

**Background:**

Attention-deficit/hyperactivity disorder (ADHD) is one of the most common neurodevelopmental disorders in childhood and is caused by both genetic and environmental factors. As genetic factors are nonmodifiable, environmental factors have attracted increasing attention.

**Objective:**

To investigate the relationships between urinary chlorpyrifos (CPF) levels, blood micronutrient levels, and ADHD prevalence in children living in rural areas of China.

**Methods:**

This cross-sectional study collected data on CPF exposure (according to urinary levels), blood micronutrient levels, and ADHD prevalence in children aged 1–6 years in rural China. The CPF levels were determined by mass spectrometry. Blood levels of micronutrients, including zinc, iron, calcium, copper, magnesium, and vitamin D, were measured by professional detection kits. ADHD was diagnosed according to the *Diagnostic and Statistical Manual of Mental Disorders*, 4th Edition. Descriptive statistics and univariate analysis were conducted using SPSS 21.0, and path analysis was conducted using Mplus 8.0.

**Results:**

Of the 738 children who met the eligibility criteria, 673 children (673/738, 91.2%) were included in the final analysis. Baseline questionnaires and urine samples were collected from all 673 subjects. A total of 672 children provided blood samples for micronutrient testing, and 651 completed the ADHD assessment. Approximately one-fifth of children (144/673, 21.4%) had detectable levels of CPF in their urine, and 6.9% (45/651) were diagnosed with ADHD. Path analysis showed that the total effect of CPF exposure on ADHD risk was 0.166 (*P *< 0.05), with a direct effect of 0.197 (*P < 0.05*) and an indirect effect of −0.031 (*P *< 0.05) *via* vitamin D. The mediating effect of urinary CPF levels on ADHD risk *via* vitamin D was 18.67%.

**Conclusion:**

Higher levels of CPF exposure are associated with higher risk of ADHD. Additionally, increasing vitamin D levels may have a beneficial effect on the relationship between CPF exposure and ADHD risk. Our findings highlight the importance of modifying environmental factors to reduce ADHD risk and provide insight into future ADHD interventions.

## Introduction

Attention-deficit/hyperactivity disorder (ADHD) is a neurological disorder characterized by persistent difficulty in regulating attention and affects 3.4 to 7.1% of school-aged children (1). Although ADHD is one of the most common developmental disorders, little is known about its underlying causes. A large number of studies have shown that the etiology of ADHD is multifaceted and encompasses both genetic susceptibility and environmental factors (2). Empirical research has suggested that exposure to heavy metals, dietary factors (3), and chemical pollutants such as bisphenol A (4), polycyclic aromatic hydrocarbons (5), and pesticides may increase the risk of ADHD. There may also be an interaction between environmental factors and genes that increases the risk of ADHD (6).

Chlorpyrifos (CPF) is one of the most widely used organophosphorus pesticides and an important environmental pollutant. Due to its persistence and bioaccumulation in the environment, exposure to CPF is unavoidable. Recently, the neurotoxicity of CPF has attracted increasing attention. The classic manifestation of CPF-induced damage to the nervous system is cholinergic syndrome, which is caused by acute poisoning and is characterized by the inhibition of cholinesterase activity. Chronic exposure to low-level CPF may have long-term adverse effects on the structure and function of the nervous system, increasing the risk of neurodegenerative diseases and neurodevelopmental disorders (7). Most previous studies have focused on the relationship between prenatal CPF exposure and offspring neurodevelopment rather than on children's direct exposure to CPF (8, 9). Although two recent studies have explored the correlation between CPF exposure and adolescent neurodevelopment, research on CPF exposure levels and ADHD in children is scarce (10, 11). As increasing evidence shows that exposure to low levels of CPF may increase the risk of ADHD, we aimed to explore this relationship in a younger group of children aged 1–6 years.

Nutrition and diet are also important environmental factors that influence the risk of ADHD (12). Recently, studies have found that increased consumption of food dyes and processed foods as well as decreased consumption of fruits and vegetables are associated with the severity of ADHD symptoms (13, 14). Follow-up studies found that many nutritional deficiencies, such as those of magnesium, zinc, iron, copper and vitamin D, are associated with ADHD symptoms (15–19). While diet therapy and nutritional supplement therapy have been explored, the results are inconsistent (20–22). More importantly, data on the relationship between micronutrient levels in children aged 1–6 years and the risk of ADHD are lacking.

To address the above two research gaps, this study aimed to investigate the relationships among urinary CPF levels, blood micronutrient levels, and ADHD risk in children aged 1–6 years. Based on the findings of previous studies, we made the following hypotheses: (1) exposure to CPF is a risk factor for ADHD; (2) micronutrients are protective factors against ADHD; and (3) micronutrients mediate the relationship between CPF exposure and ADHD risk.

## Methods

### Study population

Using cluster sampling, we selected Xinhua County in Hunan Province, Sansui County in Guizhou Province, and Gong’an County in Hubei Province to represent mountain, hill, and plains regions, respectively. In each of the three counties, three villages were randomly selected. All children aged 1–6 years in these villages were observed, except for those with the following conditions: (1) abnormal birth history, such as premature birth, multiple pregnancy, or birth asphyxia; (2) a history of head trauma; or (3) a history of serious diseases, such as genetic metabolic diseases, congenital abnormalities, feeding difficulties, severe malnutrition, recurrent respiratory diseases, or severe abnormalities of liver or kidney function. Based on the sample size recommendation for path analysis (23) and the *Medical Statistics* textbook, we determined that the minimum sample size required in each of the three areas was 200 children. We administered questionnaires, assessments of urinary CPF levels, blood micronutrient assessments, and ADHD diagnosis to all eligible children.

Prior to the field test, we verbally explained the purpose and process of the study to the parents of all children and emphasized the confidentiality of the data. The parents of all included children provided written informed consent. This study was strictly conducted according to international guidelines for the protection of human subjects and was approved by the Ethics Committee of the Third Xiangya Hospital of Central South University in Hunan, China. The ethics review number is 2014S162.

### Parameters

#### Assessment of CPF levels in children's urine

##### Collection and pretreatment of urine samples

The urine samples were collected and analyzed as follows: 20–30 ml of morning urine was collected using disposable sterile urine cups; this sample was further divided into 5-ml polypropylene bottles. Each sample was numbered and recorded. All urine specimens were sent to the laboratory within 24 h of collection and stored at −80 °C until analysis.

##### Gas chromatography–mass spectrometry analysis (GC–MS) of CPF levels

Before the gas chromatography–mass spectrometry (GC‒MS) analysis, acetonitrile was added to the urine samples, and they were vortexed for approximately five minutes. NaCl was then added, and the mixture was vortexed for approximately one minute. The supernatant was pipetted into a rotary steaming flask, evaporated until nearly dry, and then diluted to a volume of 1 ml with ethyl acetate. This solution was transferred to an Eppendorf (EP) tube with 50 mg of primary secondary amine (PSA), vortexed for 30 s, and centrifuged at 8,000 r/min for four minutes. Finally, the supernatant was passed through a 0.22-*μ*m filter, and GC‒MS analysis was performed.

Measurements of the CPF levels were performed as described elsewhere using GC‒MS analysis (Agilent GC7890A-5975). Laboratory quality control parameters for CPF levels were verified with reference to NY/T 788-2004 Pesticide Residue Test guidelines, which is the agricultural industry standard in China.

#### Micronutrient assessment

We collected venous whole blood samples from children in the morning after an overnight fast; these samples were refrigerated, transported, and stored until analysis. Measurements of micronutrients were conducted within 48 h after collection.

Vitamin D measurements were carried out using an enzyme-linked immunosorbent assay (ELISA). Monoclonal antibodies were used to detect 25-OH-D in whole blood samples with kits provided by the Oumeng Company (Germany). Calcium, iron, zinc, copper, and magnesium levels were all determined by atomic absorption spectrometry using Beijing Bohui Company's innovative photoelectric technology BH7100S. A total of 40 μl of whole blood was added to the instrument, and the samples were atomized by flame combustion, producing numerous ground-state free atoms that absorbed the characteristic spectrum of the measured elements emitted through a hollow cathode lamp. The levels of vitamin D (25-OH-D), calcium, iron, zinc, copper, and magnesium were treated as continuous variables. The blood level of lead was treated as a categorical variable (>10 µg/dl or ≤ 10 µg/dl), according to the levels of blood lead toxicity proposed by the Chinese Centers for Disease Control and Prevention (CDC). Laboratory quality control parameters for micronutrient assessment were conducted between laboratories in China every year and acquired the certificare from the national center for clinical laboratories.

#### ADHD assessment

The Diagnostic and Statistical Manual of Mental Disorders, 4th edition (DSM-IV) was used to evaluate ADHD in children aged 1–6 years. The evaluation consists of two parts, including nine symptoms of attention deficits and nine symptoms of hyperactive/impulsive behavior. An individual is diagnosed with ADHD if they exhibit six or more of the nine symptoms in one of the two dimensions and if these symptoms persist for at least six months. The ADHD outcome (with or without an ADHD diagnosis) was treated as a categorical variable.

#### Questionnaires

We used questionnaires to collect demographic characteristics and medical information from the children, such as age, sex, date of birth, primary caregiver and their educational level, annual family income per capita, maternal age at pregnancy, weight at birth, and breastfeeding history. The questionnaire consisted of a one-on-one survey with the children's primary caregivers. The investigators were uniformly trained before administering the survey.

### Statistical analysis

All data analyses were conducted using SPSS version 21.0 for Windows (IBM Corp., Armonk, NY, USA) and Mplus 8.0 (Muthén / Muthén, Los Angeles, CA, USA). The mean (standard deviation [SD]) or the median (25th and 75th percentiles [P25 and P75]) were used to describe the distribution of continuous variables based on the normality of their distribution; n (%) were used to describe categorical variables. Nonparametric tests, t tests, nonparametric tests, or chi-square tests were used for univariate analysis. Pearson or Spearman correlation analyses were then conducted to examine the correlation among all study variables. Prior to the path analysis, we used logistic regression analysis to examine whether there were significant interactions between micronutrients (i.e., calcium, iron, zinc, copper, and magnesium) and CPF levels. As shown in Supplementary Appendix 2, no significant interactions were found; therefore, no interaction terms were included in the path analysis. Significant variables (i.e., *P *< 0.05) identified in the univariate and correlation analyses and demographic characteristics related to ADHD risk (i.e., age, sex, primary caregivers, and the education of primary caregivers) were included in the next step of the path analysis (24, 25). The path analysis was conducted using the maximum likelihood method. Model fit was examined with the relative *χ*^2^ goodness-of-fit statistic (*χ*^2^/degrees of freedom [df]), comparative fit index (CFI), Tucker and Lewis's index of fit (TLI), and root mean square error of approximation (RMSEA). A model was considered to exhibit acceptable fit if it met the following criteria: *χ*^2^/df <5, RMSEA <0.08, TLI >0.90, and CFI >0.90.

## Results

### Study population and participant characteristics

**Figure 1:**
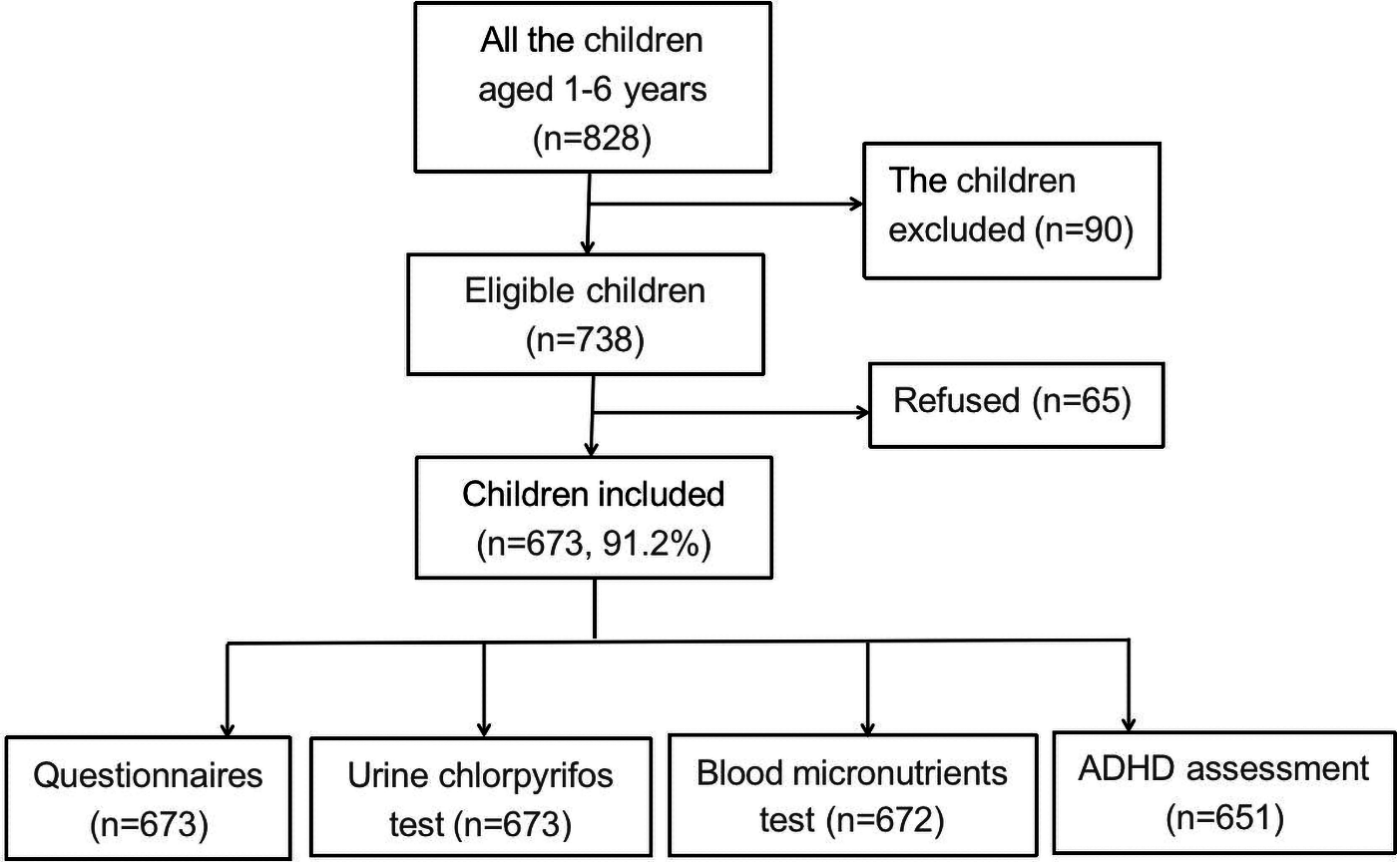
Research population and survey flow chart.

Of the 738 children who met the eligibility criteria, 673 (91.2%) were included in the final analysis. Baseline questionnaire data and urine samples were collected from these 673 subjects. A total of 672 children provided blood samples for micronutrient testing, and 651 completed the ADHD assessment (Figure 1). The mean age of the participants was 41.58 ± 15.4 months. The distributions and descriptive statistics of each variable, including sex, primary caregiver, district, education of primary caregiver, annual family income per capita, birth weight, maternal age at pregnancy, and age at weaning, are shown in Table 1.

**Table 1:** Cross-sectional survey results and ADHD univariate analysis results.

Parameters	Frequency (%) or mean ± SD/median (IQR)	ADHD		*P*
		Yes (*n* = 45)	No (*n* = 606)	
Zinc (µmol/L)	64.41 ± 9.76	64.45 ± 8.37	64.29 ± 9.83	0.916
Iron (mmol/L)	7.77 ± 0.51	7.78 ± 0.51	7.77 ± 0.51	0.930
Calcium (mmol/L)	1.69 ± 0.68	1.69 ± 0.05	1.69 ± 0.07	0.704
Copper (µmol/L)	14.14 ± 1.75	14.14 ± 1.76	14.29 ± 1.54	0.364
Magnesium (mmol/L)	1.52 ± 0.13	1.52 ± 0.13	1.54 ± 0.15	0.458
25- (OH)-D (nmol/L)	53.76 ± 8.96	50.57 ± 4.93	54.08 ± 9.25	0.000
Age of month	41.58 ± 15.4	40.40 ± 11.21	42.17 ± 15.40	0.351
Chlorpyrifos (µg/kg)	0.00 (0.00, 0.00)	0.00 (0.00, 2.80)	0.00 (0.00, 0.00)	0.176
Birth weight (kg)	3.43 ± 1.01	3.40 ± 0.93	3.44 ± 0.93	0.762
Age during pregnancy	25.19 ± 5.34	25.07 ± 5.02	25.17 ± 5.20	0.899
Weaning age	10.00 (8.00, 12.00)	11.00 (6.00, 12.50)	10.00 (8.00, 12.00)	0.867
Gender				0.505
Boy	316 (48.54%)	24 (53.33%)	292 (48.18%)	
Girl	335 (51.46%)	21 (46.67%)	314 (51.82%)	
Lead				0.718
>10 µg/dl	619 (95.08%)	1 (2.22%)	31 (5.12%)	
≤10 µg/dl	32 (4.92%)	44 (97.78%)	574 (94.88%)	
Primary caregiver				0.825
Others	356 (57.42%)	24 (54.55%)	332 (57.54%)	
Parents	264 (42.58%)	20 (45.45%)	245 (42.46%)	
Per capita income				0.004
<3,000 RMB/ year	263 (44.35%)	28 (65.12%)	235 (42.73%)	
≥3,000 RMB/ year	330 (55.65%)	15 (34.88%)	315 (57.27%)	
Education of primary caregiver				0.552
Lower than high school	495 (84.91%)	36 (81.81%)	459 (85.16%)	
High school or higher	88 (15.09%)	8 (18.19%)	80 (14.84%)	
District				0.685
Hubei Gongan	188 (28.88%)	11 (24.44%)	177 (29.21%)	
Guizhou Sansui	197 (30.26%)	13 (28.89%)	184 (30.36%)	
Hunan Xinhua	266 (40.86%)	21 (46.67%)	245 (40.43%)	

Note: SD, standard deviation; IQR, interquartile range.

### Blood micronutrient levels and urinary CPF levels

The distributions of lead levels and descriptive statistics of each variable, including levels of zinc, iron, calcium, copper, magnesium, and 25-(OH)-D, are shown in Table 1.

**Figure 2:**
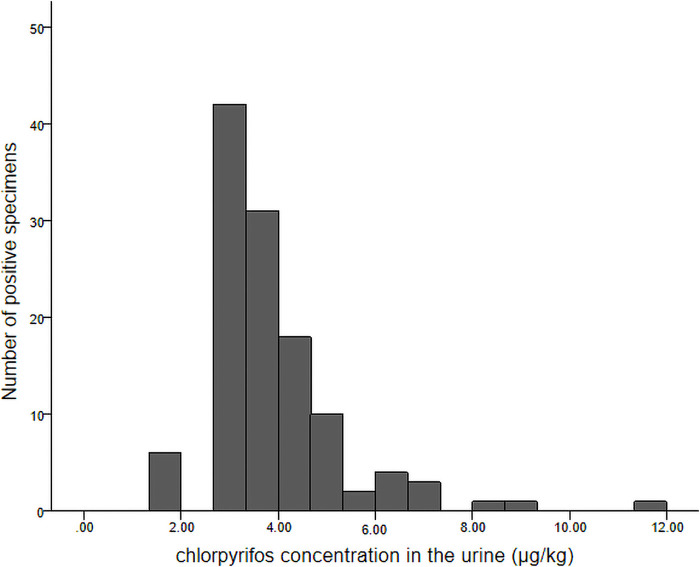
Histogram of chlorpyrifos concentrations detected in urine samples and the corresponding number of positive samples.

Approximately one-fifth of children (144/673, 21.4%) had detectable levels of CPF in their urine. The number of positive samples and their corresponding CPF concentrations are shown in a histogram (Figure 2).

### Results of ADHD assessment and univariate analyses

In total, 45 children were categorized as having ADHD, a positive rate of 6.9% (45/651). As shown in Table 1, there were significant differences in annual family income per capita and vitamin D levels between children with ADHD and those without ADHD. Specifically, children had a higher risk of ADHD if they had lower vitamin D levels and came from lower income families.

### Results of the correlation and path analyses

The correlation results showed that vitamin D levels and age in months were positively correlated with CPF exposure, that annual family income per capita and vitamin D levels were positively correlated with ADHD risk, and that primary caregiver education was positively correlated with vitamin D levels (*P *< 0.05; see Supplementary Appendix 3).

**Figure 3:**
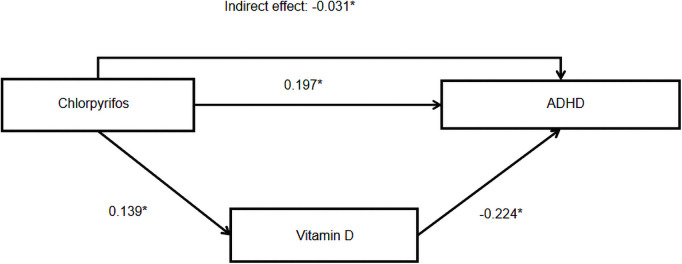
Model linking chlorpyrifos exposure to ADHD. Note: * indicates *P* < 0.05, statistically significant effects are indicated by solid lines.

The path model showed good fit, as shown by the normed chi-square value of 2.37, RMSEA of 0.048, TLI of 0.901, and CFI of 0.984. As shown in Table 2 and Figure 3, CPF had a direct effect on ADHD risk (estimate = 0.197, *P < 0.05*) as well as an indirect effect *via* vitamin D levels (estimate = −0.031, *P < 0.05*). The mediating effect of urinary CPF levels on ADHD risk *via* vitamin D was 18.67%.

**Table 2:** Standardized effect size of associated factors of ADHD analyzed using path analysis.

Variable	Estimate	Standard error	*P*
Total effect CPF → ADHD	0.166	0.070	0.018
Direct effect CPF → ADHD	0.197	0.071	0.006
Total indirect effect CPF → ADHD	−0.031	0.015	0.048
CPF → Vitamin D	0.139	0.040	0.000
Vitamin D → ADHD	−0.224	0.101	0.018

Note: Toltal effect size = Direct effect size + Indirect effect size.

## Discussion

In the present study, we investigated the relationships among urinary CPF levels, blood micronutrient levels, and ADHD risk in rural Chinese children aged 1–6 years. Consistent with our hypotheses, we found that (1) CPF exposure was a risk factor for ADHD; (2) higher levels of vitamin D may reduce the risk of ADHD; and (3) vitamin D levels mediated the relationship between CPF exposure and ADHD risk.

This cross-sectional study investigated CPF levels in children's urine and found that 21.4% of children had detectable CPF levels. In 2016, a cross-sectional study of 140 children living near a banana plantation in Talamanca, Costa Rica reported that 40.7% (57/140) of children had urine samples positive for the CPF metabolite 3,5,6-trichloro-2-pyridinol (TCPy) (11). In addition, a cross-sectional survey of 60 children (aged 1–6 years) of North Carolina farm workers found that 83.3% of the children had detectable levels of CPF or CPF-methyl in their urine (26). Furthermore, 230 residents of Yunmou County, China underwent testing for urinary CPF; the positive rate was 10%. These results indicate that CPF exposure is related to the environment in which the participants live as well as the methods used to assess their exposure. Furthermore, knowledge of pesticide use influences children's pesticide exposure, indicating the importance of caregiver awareness (27).

The ADHD assessment revealed that 6.9% of children in rural China had ADHD in our study, a rate similar to that in previous studies of children in mainland China (6.3%) (28). Our study indicated that exposure to low-level CPF in the environment may increase the risk of ADHD in children aged 1–6 years. Previous studies showed that maternal exposure to CPF are related with ADHD in children (8, 9). Yu et al. reported that a dose-response relationship was found between urinary concentrations of organophosphate pesticide in Taiwanese children aged 4–15 and ADHD (29). Moreover, mouse experiments have also shown that prenatal CPF exposure impacts the motor activity and memory of offspring (30). But data on the relationship between CPF residual levels in children aged 1–6 and the risk of ADHD are lacking. As a multi-center cross-sectional study, our study is more conducive to early detection, diagnosis, and interventions of ADHD by screening ADHD among preschool children. The mechanism underlying neurotoxicity induced by exposure to low-level CPF remains unclear. Some studies have suggested that exposure to low levels of CPF affects motor coordination and function by influencing extracellular GABA concentrations in the cerebellum as well as NMDA receptor subunits in the hippocampus (30). Perinatal exposure to pesticides, such as CPF, is also correlated with alterations of synaptic plasticity that impair brain development and synaptic signaling (31). In addition, CPF exposure can interfere with transglutaminase activity in tissue as well as transient potential channels (32, 33). CPF-induced oxidative stress and its concomitant damage, including oxidative injury, metabolic imbalance, and lysosomal damage, are also likely correlated with its neurotoxic effects (34). However, counter to the mounting evidence of the neurotoxicity of CPF, researchers reported that early exposure to CPF in rats does not inhibit AChE activity in the blood or brain (35). Thus, further research is needed to elucidate the mechanism underlying neurotoxicity induced by exposure to low-level CPF.

This study also investigated micronutrient levels and their relationships with CPF exposure and ADHD risk. This design introduces biological samples (urine and blood samples), which are more direct and consequential for verifying the impact of environmental factors. Univariate and multivariate analyses revealed that vitamin D levels have a protective effect against the risk of ADHD, a finding consistent with the results of previous studies. Accumulated data suggest that decreased levels of vitamin D may also be a significant risk factor for ADHD (36–38) and that increased prenatal levels of vitamin D may have a protective effect against ADHD (39). However, a Danish cohort study and a Swedish case‒control study found no association between vitamin D levels and ADHD in older children (40, 41) Further research is necessary to determine whether an adequate supply of vitamin D can protect against ADHD. Regarding the underlying mechanism, vitamin D is a steroid hormone and can freely cross the blood‒brain barrier, thereby influencing neurons and glial cells. Research has indicated that microglia produce calcitriol *in situ*, upregulating the synthesis of nitric oxide synthase by glutathione; nitric oxide synthase has neuroprotective and neuromodulatory functions (42–44). Additionally, studies have suggested that vitamin D protects against many pathophysiological processes of neurons, such as oxidative stress as well as impaired neuronal calcium homeostasis, nerve conduction, detoxification, and immune regulation (45). Furthermore, vitamin D interacts with the promoters of various receptors, exerting epigenetic effects and thus altering their function (46–48). Therefore, vitamin D deficiency has the potential to promote neurodevelopmental disorders such as ADHD.

Furthermore, path analysis showed that CPF exposure was significantly correlated with vitamin D levels. The interaction between CPF and Vitamin D was studied in lab experimental setting. Krzysztof et al. reported that CPF can interfere with vitamin D3 metabolism in skin cells by altering the expression of the vitamin D3 receptor and enzymes such as CYP27A1, CYP27B1, and CYP24A1 (49, 50). As to the relationship of CPF exposure in children with vitamin D, little studies can be found on. This is the first cross sectional study that found the possible correlation between them. The skin is regularly exposed to environmental pollutants, including CPF; it is also the organ responsible for vitamin D3 synthesis. Thus, the interaction between CPF and vitamin D also warrants attention from the perspective of epidermal cells. However, studies on the interplay between CPF and vitamin D are scarce, and further research is needed.

This study has several limitations. First, our study was based on cross-sectional data. Further research using a longitudinal design is needed to confirm the causal relationship between CPF exposure and ADHD risk. Second, there may be self-report bias regarding ADHD. Future research should consider using different forms of data collection to verify whether such measurement bias exists. The third limitation of our study is the possibility of residual confounding, which can not be fully eliminated in an observational study. For example, in this study we did not include children's allergy history and eating behaviors, which may result in biases in our analyses. These variables should be taken into consideration in future studies (51, 52). Finally, using a one-time measurement of urinary CPF levels may not be the best method for estimating long-term CPF exposure; thus, a follow-up study is necessary.

In conclusion, we investigated the relationships between CPF exposure, ADHD risk, and micronutrient levels in rural Chinese children aged 1–6 years. We found that exposure to CPF was a risk factor for ADHD in children and that higher levels of vitamin D protected against ADHD. Supplemental vitamin D for children may thus help protect against ADHD risk in children exposed to CPF. Our findings provide insights for future ADHD interventions that involve modifying environmental risk factors, such as reducing CPF exposure and increasing vitamin D intake, to reduce ADHD risk.

## Data Availability

The original contributions presented in the study are included in the article/**Supplementary Material**, further inquiries can be directed to the corresponding author/s.
