# Supplementary material for: Chlorpyrifos residue level and ADHD among children aged 1–6 years in rural China: A cross-sectional study
Source: Front Pediatr. 2022 Oct 14;10:952559. doi: 10.3389/fped.2022.952559 (PMC9616114; doi:10.3389/fped.2022.952559)
Supplement: Supplementary file 1 [file Table2.docx]

**Appendix Table 2** Bi-variate correlation of variables

| Variables | CPF | Vitamin D | ADHD |
| --- | --- | --- | --- |
| CPF | 1.000 |  |  |
| Vitamin D | 0.141* | 1.000 |  |
| ADHD | -1.315 | 3.898* | 1.000 |
| Age of month | 0.106* | -0.057 | 1.501 |
| Gender | 1.827 | 1.711 | 0.220 |
| Primary caregiver | -0.260 | 1.797 | 0.090 |
| Education of primary caregiver | 0.750 | 3.330* | 0.309 |
| Per capita income | -0.772 | -1.478 | 8.100* |

**P* < 0.05.
