# Supplementary material for: Chlorpyrifos residue level and ADHD among children aged 1–6 years in rural China: A cross-sectional study
Source: Front Pediatr. 2022 Oct 14;10:952559. doi: 10.3389/fped.2022.952559 (PMC9616114; doi:10.3389/fped.2022.952559)
Supplement: Supplementary file 2 [file Table3.docx]

| Variables | Score | df | Sig. |
| --- | --- | --- | --- |
| CPF | 1.791 | 1 | .181 |
| Zinc | 0.011 | 1 | .915 |
| Iron | 0.008 | 1 | .929 |
| Calcium | 0.145 | 1 | .704 |
| Copper | 0.374 | 1 | .541 |
| Magnesium | 0.407 | 1 | .524 |
| Age of months | 0.575 | 1 | .448 |
| Gender | 0.445 | 1 | .505 |
| CPF*Zinc | 1.824 | 1 | .177 |
| CPF*Iron | 1.871 | 1 | .171 |
| CPF*Calcium | 1.873 | 1 | .171 |
| CPF*Copper | 1.871 | 1 | .171 |
| CPF*Magnesium | 1.942 | 1 | .163 |

Appendix 2 The interaction between micronutrients and chlorpyrifos.

Note: Forward conditional logistic regression method was used for analysis
