# Supplementary figures and images for: Chlorpyrifos residue level and ADHD among children aged 1–6 years in rural China: A cross-sectional study
Source: Front Pediatr. 2022 Oct 14;10:952559. doi: 10.3389/fped.2022.952559 (PMC9616114; doi:10.3389/fped.2022.952559)

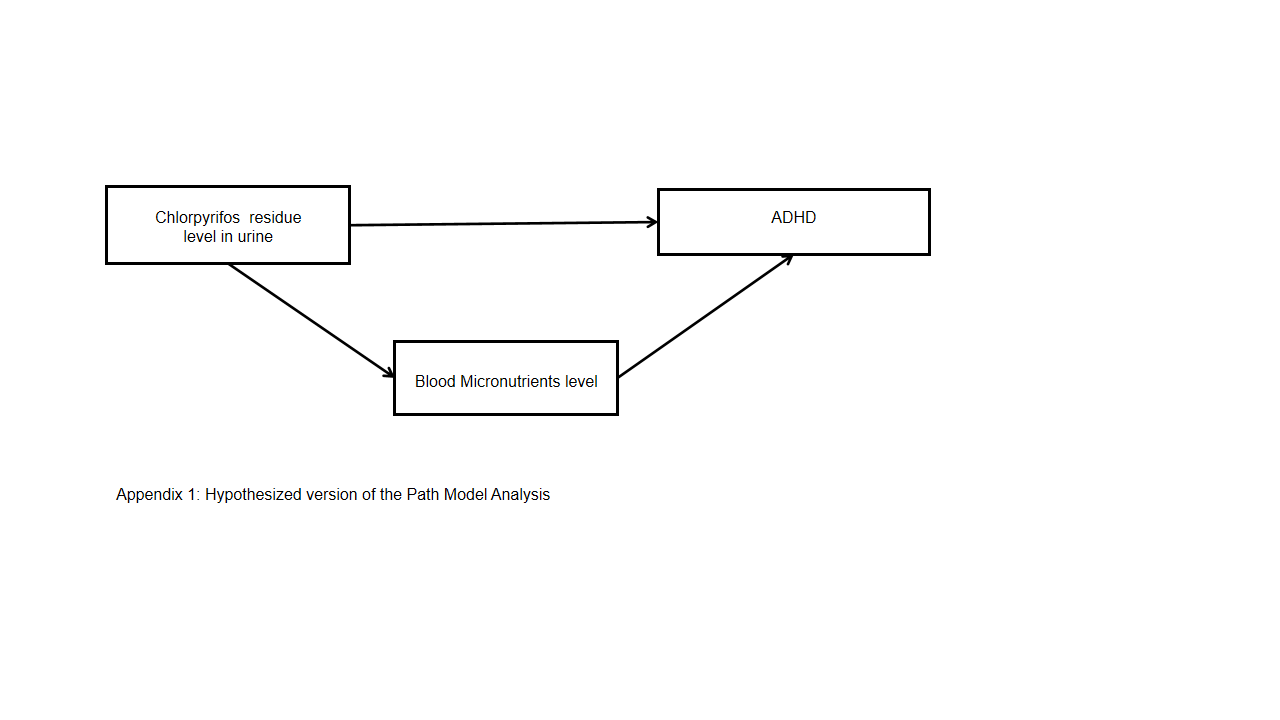

Supplement: Supplementary file 3 [file Image1.tif]
